# Relationships between community-led mutual aid groups and the state during the COVID-19 pandemic: complementary, supplementary, or adversarial?

**DOI:** 10.1080/14719037.2022.2084769

**Published:** 2022-06-06

**Authors:** Jack Rendall, Maeve Curtin, Michael J. Roy, Simon Teasdale

**Affiliations:** The Yunus Centre for Social Business & Health, Glasgow Caledonian University, Scotland

**Keywords:** Mutual aid, public health, civil society, public management

## Abstract

This research explores ways public service ecosystems developed during the COVID-19 pandemic, focusing on relationships between community-led mutual aid groups and the state. Data were collected through in-depth interviews, focus groups, and mobile ethnographic methods with 30 participants from the public sector and three mutual aid groups across Scotland. We show how relationships between mutual aid groups and the state – whether complementary, supplementary, or adversarial – shifted over the course of the pandemic. Our findings add nuance to understandings that presuppose mutual aid as antagonistic, highlighting ways that mutual aid groups may be brought into existing public service ecosystems.

## Introduction

The global COVID-19 pandemic has disrupted the social networks we rely on … COVID-19 represents a major rupture in the status quo and calls for new forms of response. Perhaps this is why thousands of new “mutual aid” groups have sprung up internationally (Mahanty and Phillipps 2020, 1).

The community response to the COVID-19 pandemic has been vast. More than 4,000 local solidarity networks describing themselves as ‘mutual aid groups’ (O’Dwyer 2020; Cooney 2020) involving tens of thousands of people, emerged organically across the UK over a matter of days (Booth 2020). Mutual aid is when people come together to ‘support, help and influence each other in a reciprocal manner … ’ (Gitterman and Schulman 2005, xi). Providing local community support to those most at risk of the effects of the virus, such as elderly people, disabled people, and those with other pre-existing health issues (COVID-19 Mutual Aid UK 2020), many COVID-19 mutual aid groups in the UK organized themselves on a hyper-local basis ‘with the smallest consisting of just a handful of members and the largest comprising more than 1000’ (Cooney 2020, 1).

In this paper, we report findings from a mixed-method study exploring how relationships between mutual aid and existing public service ecosystems in Scotland evolved over the course of the pandemic. Relationships between the state and civil society have been characterized in the past as *supplementary*, *complementary*, or *adversarial* (Young 2000). Drawing on this framework, our paper is organized as follows: after a discussion of the key background literature that frames our study, we describe the research methods we employed and provide relevant background on the three different settings in which each mutual aid group we studied is embedded. We then outline our findings and discuss these in relation to the dynamics of public management and civil society responses during periods of crisis. First, we turn attention to what we mean by ‘mutual aid’ and the question of why it is important to understand relationships between civil society organizations and the state.

## Background

Community responses to crises are not a new phenomenon by any means (Solnit 2009), and mutual aid groups have emerged in response to crises in the past (Kenney 2019; Spade 2020). Kropotkin's (1902) seminal book *Mutual Aid: A Factor of Evolution* recognized that the most effective communities of humans and other species are ones that are essentially cooperative, rather than competitive. Since the early medieval craft guilds, mutual aid has been institutionalized in the form of trades unions and the friendly and mutual societies that became commonplace throughout Europe during industrialization. American fraternity societies emerged during the Great Depression, supporting members and their families with health insurance and funeral and life assurance benefits. The English working men’s clubs of the 1930s also provided health insurance benefits (Spade 2020).

Numerous examples of mutual aid groups emerging in response to environmental, social, and economic crises in the past have generally been in cases where support from the public sector has been insufficient, ineffective, or slow to mobilize (Michael et al. 1985). For example, the mutual aid group Occupy Sandy emerged in the immediate aftermath of superstorm Sandy in 2012 in New York:

In many cases, Occupy activists were playing the role of first responder – particularly to those residents living in public housing and racialized enclaves in Queens and Brooklyn, which were effectively off the map of state-led recovery efforts (Conroy 2019, 876).

According to Mutual Aid Disaster Relief, which was formed in 2005 based on the concept of ‘solidarity not charity’ in the aftermath of Hurricane Katrina, the role of mutual aid groups in the face of the novel coronavirus outbreak is:

Piecing together carefully constructed information on defending our communities, building bridges over access and info gaps for folks with differing vulnerabilities and sharing comprehensive information on harm reduction, DIY resource building and responding with best practices … (Mutual Aid Disaster Relief 2020).

While we know how mutual aid groups emerge in response to crises, particularly where the state response is lacking, or insufficient, rather less is known about how they evolve over time, particularly once government actors return to these institutional voids. Relationships between civil society organizations and the state tend to be highly complex, and fluid (Young 2000, 2006; Bode and Brandsen 2014). Relationships play out in a ‘complex and dynamic ecosystem’ formed by ‘various nested layers of interaction’ (Strokosch and Osborne 2020, 429). These interactions may be adversarial or cooperative, or both at the same time (Laamanen and Skålén 2015). As with a natural ecosystem, an exogenous shock (such as Hurricane Katrina, or COVID-19) can expose weaknesses in a public service ecosystem. Following COVID-19, those with similar ideas about what would best help communities respond and sustain themselves throughout the crisis formed mutual aid groups to address what their members perceived as gaps in public service delivery, presenting challenges to the existing ecosystem in different ways. To explore the relationships between various actors in detail, and how these developed over time, it is helpful here to draw upon Young (2000) three categories describing relationships between civil society and the state: *supplementary*, *complementary*, or *adversarial*.

*Supplementary* denotes a role whereby civil society works to fulfil the demand for public goods left unsatisfied by the state: ‘As government takes more responsibility for provision, less needs to be raised through voluntary collective means’ (Young 2000, 150). Based on the premise of state – as opposed to market – failure, this perspective was first observed in the 1980s Reagan administration, which was intent on rolling back government spending while imagining that civil society would simply fill the vacuum caused by the retreat of the state through volunteer efforts and philanthropic donations (Young 2006). Such an ‘oversimplified’ view of civil society-government relations has been regularly apparent in the UK, re-emerging sharply post-Global Financial Crisis, underpinning much of the ‘Big Society’ thinking, where state responses were considered to crowd out voluntary action (see Alcock 2010; Teasdale, Alcock, and Smith 2012).

Research has long examined the role of civil society actors acting *complementary* to the work of the public sector, regularly framing civil society actors as effective partners for public service delivery (e.g. Brandsen and Pestoff 2006). Commonly, such services have come to be designed and/or delivered through a ‘co-production’ arrangement (Nabatchi, Sancino, and Sicilia 2017; Bovaird 2007) where civil society actors have started to be included in policy development discussions, seen as potential partners for designing and commissioning – in addition to simply delivering – public services (Brandsen, Steen, and Verschuere 2018). The role of the state and civil society in co-producing policy responses has reinvigorated scholarship around public sector and civil society collaboration, exemplified in the idea of New Public Governance (Osborne 2006), which emphasizes collaborative policymaking and opportunities for collective decision making. Collaboration is considered to improve public policy outcomes (Vaillancourt 2009), democratizing relationships with civil society by allowing citizens to engage as co-producers and serve as active stakeholders in service delivery (Mazzei et al. 2020; McMullin 2020). ‘From the perspective of government’ argue Bode and Brandsen (2014, 1056) ‘non-public partners are very much welcome as they are expected to enrich the repertoire of public management and to provide relief to an ever more disarmed welfare state’.

The third role – *adversarial* – is when civil society identifies government policies or resource allocation strategies which do not match the needs of society, and then strives to change them (Derrick-Mills 2015). Such struggles are a central premise of social movement scholarship (Della Porta et al. 2020) and there are innumerable examples of social movements mobilizing civil society to address structural inequities. While mutual aid groups are regularly characterized as being adversarial, towards both the state and the idea of traditional charity (Dayson and Damm 2020), such an adversarial relationship need not be thought of as altogether negative since antagonism can be a positive force for change (North 2020).

While the literature around mutual aid responses to the COVID-19 pandemic has barely started to emerge, reports and articles have begun to stress the importance of, and the complexities of, understanding partnership working in such settings (e.g. TSI Scotland Network, and Evaluation Support Scotland 2021). Meanwhile, international work has shown the power of informal networks and collective action in addressing inherent inequalities, and how community-level responses interface with top-down ways of working in innovative and complex ways. Such work highlights the productive potential for developing understanding of how public services can better engage with more antagonistic forms of collective action without jeopardizing the potential of community efforts (van Ryneveld, Whyle, and Brady 2020, 1). We next turn attention to the methods employed in our study.

## Methodology

Mutual-aid work thrives on sustained personal relationships, but the coronavirus has necessitated that relationships be built online (Tolentino 2020, 1).

This research was designed to be undertaken very rapidly at the height of the crisis, with the broad goal of understanding how mutual aid groups were responding to the crisis. It was one of a select group of research studies funded at that time by the Scottish Government Health Directorate’s Chief Scientist Office, and methods had to be attuned appropriately and the study set up and implemented quickly, to cope with (what was then) an indefinite lockdown. Given the infeasibility of conducting in-depth face-to-face meetings or group observations during that time, we employed digital ethnographic methods (Akemu and Abdelnour 2020), complemented by video-recorded, semi-structured interviews. Digital ethnography ‘creates deep, contextual and contingent understandings produced through intensive and collaborative sensory, embodied engagements, often involving digital technologies in co-producing knowledge’ (Postill and Pink 2012, 125). For this study, we developed several online activities designed on a web-based platform called *Recollective*. These activities enabled the gathering of real-time perspectives from individuals involved with mutual aid groups while they were actively engaged with the groups’ work; data gathered reflected their opinions without reliance upon memory or the influence of hindsight. Examples of these activities included prompts to upload and discuss photos, videos, and screenshots related to various mutual aid group impacts, and a card sorting activity to determine attitudes towards formal public services and providers. 20 participants submitted 165 unique interactions, 56 unique impact examples, 13 photos and four videos.

Data collection took place from mid-June to mid-September 2020, but participants shared and reflected upon their own personal and organizational experiences dating back to March 2020. Towards the end of this period, we began conducting online semi-structured interviews (n = 20) with participants. Insights previously gathered from the online activities and tasks provided us with the opportunity to understand the lives of participants at a deeper level than might have otherwise been possible without the digital methods. As Tremblay et al. (2021) have noted, digital methods can add rigour and reliability exceeding that typically associated with face-to-face interactions because participants may experience feelings of increased control and comfort, coupled with fewer inhibitions, when sharing personal thoughts and emotions. In the context of this study, it enhanced the depth of each interview and the focus group discussions because we prompted participants to elaborate on insights and experiences they shared previously.

### Group sampling

Scotland has 32 unitary local authorities (or ‘councils’ – we use the term interchangeably, as did our research participants) which provide public services, including education, social care, waste management, libraries, and planning. Each operates independently of the devolved Scottish Government and is accountable to their electorates for the services they provide. Third sector/community organizations are represented in the ‘Community Planning Partnership’ for each local authority area by a Third Sector Interface (TSI), a model that is designed to encourage partnership working across sectors. Given that each mutual aid group was autonomous, varying in their composition, size, and activities due to the geographies they covered, we decided to examine one group in three different local authority areas in Scotland – one each in an urban, peri-urban, and rural setting – to explore the importance of context. We determined which groups we should engage with after preliminary contact through Facebook Messenger and email. The final decision was based on their difference to one another to achieve a level of heterogeneity, as well as difference in terms of their activities, structure, and size.^1^ All three groups self-identified as mutual aid groups on their public-facing media.

### Case descriptions

Next, we describe each group as we found them during our recruitment phase, and while these organizations all changed in various ways over the course of the pandemic, the differences outlined below largely remain. It is therefore important to highlight these areas as they are referred to during the analysis section. Each case was given a pseudonym to help protect the identity of participants.

#### Case 1 – the green isles

A rural island group based to the north of the Scottish mainland, with a population of around 22,000 people. The group formed via Facebook on 14 March 2020. Their membership at the time of recruitment for this study was 2,053 with seven administrators for the page. 13 smaller sub-groups formed from this main group to cover parishes within the county. They used a Facebook page to share information including: opening hours changing; businesses offering deliveries or closing altogether; updates from the local authority and NHS; stories from the media; requests for help, or requests for advice and information.

#### Case 2 – the dockyards

A peri-urban group based in west of Scotland, where the population is around 78,000 people. Founded on 13 March 2020, this group was initially a strategic working group of smaller formal civil society organizations who began collaborating to meet needs more effectively across the community. Their aim was to prevent duplication of work across the region so they would coordinate services to assist vulnerable groups within their region. By 18 June 2020 the group represented 10 civil society organizations, over 400 volunteers, and had 11 different coordinated services operating across the community. Many, although not all, of the most active coordinators in the group came from already established organizations in the community and engaged with this group’s work on top of, but still within, their formal responsibilities.

#### Case 3 – the bustle

The third group was based in Scotland’s largest city, where the population is around 600,000 people. The group started on Facebook on 14 March 2020. Membership on their Facebook page was around 3,000 people at the time of recruitment for this study. Since the group covered a large geographic area with a high population density for Scotland, there were five sub-groups organized by postcodes and neighbourhoods. The group was structured in its approach: a full group meeting took place within three days of the group’s formation. Members organized various working groups to support different services (e.g. phone lines, deliveries, support calls) and work on various projects (e.g. research, skill share sessions). The group utilized online messaging software including WhatsApp and Slack.

### Participant sampling

Individuals were sampled purposively (Mason 2002) to gain the views of especially ‘data rich’ participants. These individuals included the ‘gatekeepers’ but also, due to the relatively flat hierarchy of the mutual aid groups, individuals with varying responsibilities to ensure an accurate representation of both the groups and their activities. Only 28% of our final participant pool identified as male: this balance is typical within civil society work around social care, where women disproportionately engage with less formal work (see Teasdale et al. 2011). Further, this gender split also aligns with our focus group sub-sample where 30% of participants identified as male.

We recruited 39 participants to sign up to *Recollective* and participate in the activities and interviews, but there was a high dropout rate, with 20 participants engaging with activities and completing interviews. We expected a dropout rate of 50% so we oversampled to compensate. This dropout rate is consistent with other similar studies conducted during the COVID-19 pandemic (Sevelius et al. 2020). Throughout the data collection process, the personal circumstances of individuals often changed rapidly, as did their motivation to engage with mutual aid group activities in general, following trends observed in other countries like Denmark where volunteer engagement declined as lockdowns lifted and people’s perceived responsibility to their communities decreased (Toubøl et al. 2022). This meant data from some individuals were captured over a couple of days, while others who were more engaged were able to spread their time across the three months. Beyond the participants from mutual aid groups, we also recruited ten individuals to participate in two focus groups (n_1_ = 4, all public sector representatives + n_2_ = 6, all formal third sector representatives). These focus groups were conducted with individuals from formal public health services, civil society, local government bodies, and other established voluntary organizations. The real names of individuals and organizations have been disguised using pseudonyms, in accordance with the ethical approval we received from the University for this study.

### Method of analysis

Multiple sources of qualitative evidence and several kinds of data (from 20 interviews, observations by participants including impact examples, online activity responses (the activities are outlined in the methodology section), documents provided by the mutual aid groups, and three hours of ethnographic observations of two online mutual aid group meetings) were brought together, transcribed, coded thematically (Braun and Clarke 2006) and triangulated to develop coherent preliminary findings. This approach to analysis follows the framework to enhance qualitative rigour suggested by Gioia, Corley, and Hamilton (2013) whereby first order terms (informant-centric) are found within the data then organized into second order codes (theory-centric) before they are then gathered into more coherent groups of themes. One of the most interesting and relevant informant-centric themes we identified concerned the differences in relationships between the mutual aid groups and the public sector. To explore this aspect in greater detail, we revisited our data and salient extant literature on state/civil society relationships, which provided us with a framework to construct our second order codes into our three themes (that is: whether we were seeing supplementary, complementary, or adversarial types of relationships in each context) following the categories presented by Young (2000) .^2^ Our analysis at this point also revealed that these positions were not static; they changed over time. We therefore re-analysed our data by period to reveal the dynamic nature of these relationships over the early phases of the crisis as it was unfolding, allowing us to construct our case histories. Despite the study not originally being designed to learn more about relationships between mutual aid groups and the public sector/formalized civil society, since this theme emerged most prominently from our data, we wanted to discuss these preliminary findings through focus groups. Towards the end of the study we set these up to further enhance the validity of our findings through a cross-examination by experts across different fields (Altheide and Johnson 1994).

To support understanding, our methodology – including our iterative approach to analysis – is displayed via a figure in Appendix S1. In the next section we focus on the findings from this analysis, using quotes from interviews, focus groups, and online activities as our primary evidence. Examples of additional ethnographic observations that were used to inform our overall understanding of the dynamics in each case are included in Appendix S2.

## Findings

Analysis of our data revealed a complex interaction between mutual aid groups and local formal services that evolved over time with the pandemic. Importantly, the evolution of these relationships was occurring within an extensive ecosystem defined by a broader set of governance arrangements and other ongoing responses to the pandemic. As such, this section outlines the evolution of the groups, both externally and internally, in terms of group operations and organizational structures, as they responded to programmes and services developed by formal organizations and the public sector. After an initial discussion of the different stages, we show the predominant position of each group at each stage of their pandemic response.

### Stages of change

**Figure 1.**
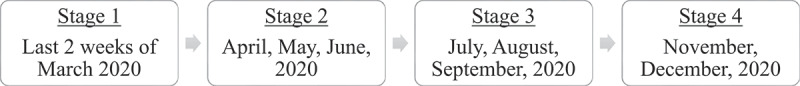
Mutual aid.

This study covered a time when four stages of organizational pandemic response emerged (see Figure 1). These stages also corresponded to shifts in the pandemic across Scotland, so as the needs of the community changed according to the changing restrictions, so too did the mutual aid groups and their relationships with formal organizations.

#### Stage 1

The relative haste with which the mutual aid groups provided support for vulnerable and shielding individuals in comparison to the state characterized this stage. For example, across all three cases, there was little to no provision of medical prescription collection and delivery by state providers. Mutual aid groups were able to provide this service even before the beginning of the national lockdown. In contrast to this, the public sector, while delivering some limited more traditional services, were still in the process of setting up support arrangements according to advice from the Scottish Government.

***The Green Isles – Stage 1.*** Almost immediately after the announcement of the lockdown, the Green Isles began to offer support for the community. This support was largely in the form of dropping off or picking up food and/or prescriptions, as well as smaller tasks such as walking dogs or posting items. During this initial stage, the support from the group was one of the only widespread offerings of support for the community. Annie, a member of the Green Isles reflected on the help they provided to vulnerable people during this stage through a journal entry: ‘I wonder how they would have managed, especially in the early weeks before the council arrangements were in place’. Once these services were set-up, some Green Isles members perceived local authority services to only serve those on the official shielding list, not those who were otherwise vulnerable to the impact of the pandemic restrictions such as those who were unemployed or lone parents.

***The Dockyards – Stage 1.*** Initially the Dockyards group created and provided a food box service for those self-isolating or shielding, as well as providing a daily contact telephone service that had made over 1,000 calls at the time of recruitment for this study. They also provided a prescription pick-up and delivery service, provided support boxes for those in the hospital. Callum, an organizer within this group revealed that in the early stages, it was not just the public sector whose response was perceived as slow, but that Third Sector Interfaces (TSIs – a single point of access for support and advice for the third sector within local areas) and coordinating bodies ‘still couldn’t get themselves organized into the lead – events overtook them and much like the council, [they] were playing catch-up from that point onward’.

***The Bustle – Stage 1.*** Services initially provided by the Bustle group included: distribution of food and medicine, training for volunteers, and mental health support for the community. In one of the online activities completed by Danielle, one of the Bustle’s coordinators, she revealed that early in Stage 1, people came to them requesting help and noting that the local authority had ‘not been clear with people on what shielding support [they would provide]’ for people who were told that they had to self-isolate due to being in the most at-risk category. Some of the requests for help were difficult for the mutual aid group to deal with because they were sometimes a last resort for people that had been let down by their local authority. In Caroline’s interview she reflected that people within the Bustle were very reluctant to say ‘no’ to anyone:

… you get stuff coming through and people were like ‘I’ve got no food for my three-year old for tomorrow’ and it would be like 7pm on a Friday night … I think people involved in the mutual aid [group] were like, ‘right, we must go to the ends of the earth to get [them] that food’ … whereas people working in the [public] sector may have been like ‘you got in touch too late’

Because of this perceived dearth of support from the local authority, members of the mutual aid group explained how this confirmed the need for the group. It should be noted that, from the perspective of those in the existing public service ecosystem, this demonstrated something of a lack of awareness of what was going on elsewhere. As Bonnie, who works in the public sector, explained during a focus group, mutual aid groups in this area ‘were replicating existing services without finding out what was already happening’. Despite this, the Bustle group maintained that at least in this early stage, while there may have been formalized services that resembled their activities, they were largely inaccessible or too inconvenient to serve community needs during that period.

#### Stage 2

During this stage, beginning approximately two weeks after the start of the lockdown, the public sector started to build up their own support systems. Mutual aid groups adapted, from being the primary service provider, towards addressing more specific individual needs not covered by more uniform public sector responses. Participants gave examples of responding to requests for delivering small quantities of food, replacing light bulbs, taking rubbish bins out, and other similar services.

***The Green Isles – Stage 2***. The Green Isles group began to gain some recognition from the local authority for the mutual aid group’s work at the local level, down to parish areas and neighbourhoods. In an interview, one mutual aid group member, Helen, recounted a situation where a local woman had requested help from the local authority, but they did not know where she lived and so were unable to help her effectively. The local authority staff member then messaged Helen to see if she would be able to help since she was the representative for that parish.

This example highlights the change in type of relationship compared to the first stage where the mutual aid group was acting in a supplementary role and had little to no interaction with the public sector. While the local authority was still not providing the service in this example, it does show an instance of complementary collaboration that was repeated more frequently in this stage.

***The Dockyards – Stage 2.*** In The Dockyards, service delivery continued in a similar manner to Stage 1; however, by this period the local authority had started to organize and deliver services. Elizabeth, one of the organizers from The Dockyards, reflected through an online activity that the local authority began ‘wrangling over petty issues and nit-picking in the face of extreme need’. She perceived that the local authority was therefore slowing down the impact that services could have on the community compared to the initial stages of the group’s operation where they were not ‘hampered’ by the local authority. George, another member of this mutual aid group, reflected through an online discussion that:

Local councils have not taken the chance to really support local communities. They have thrown some resources about, but it is local communities and volunteers that have really delivered the immediate and essential support required in an emergency. Hopefully they can learn from this and restructure their organizations accordingly.

From the perspective of formal organizations, representatives expressed levels of apprehension around the risks of personal protection of both the mutual aid group members and those who requested support from the groups. When interviewed, one volunteer manager, who was not part of a mutual aid group, whom we call Lisa said:

I think there’s a very fine line between that sort of informal approach and needing something which requires more paperwork being filled out … I know people just want to help, they don’t want to sign up to anything. And that is the niche that those groups fill. But doing more than those low-level activities then they might have to re-think what they’re doing …

***The Bustle – Stage 2***. The Bustle group was very organized in the early stages and were able to coordinate multiple different services that continued in to Stage 2. However, some members noticed at this stage that they were supporting people who may otherwise have been helped by more formal services such as from the public sector or large charities. There was a perception that people were coming to the Bustle even though other formal services were beginning to operate again. In an interview with Caroline, one of the Bustle’s coordinators, she explained that from her perspective:

… there was a perception of mutual aid that it was maybe less condescending, maybe because it hasn’t had time to build up a lot of the stigma that say food banks do … it was just the idea of we’ll just connect you up with someone who will help you out in whatever way you need and it’s not necessarily doing it as a … mutual aid volunteer, it’s just doing it as like your neighbour or another randomer …

There were some initial concerns from the local authority that the Bustle were not equipped to cope with complex community needs at this stage. This contributed to a more adversarial relationship. Focus group participants such as Rodger (Public Sector) argued ‘it is very difficult for mutual aid groups to come through … because these [substance abuse and domestic violence] are complex issues’. Caroline, a coordinator for the greater Bustle group, noted that despite these concerns, they often ‘had requests referred to [them] from the local council/social services, with no corresponding material support to do the work, and often for people with very complex care needs … ’. In these cases, the frustration from mutual aid groups seemed to be around lack of resource allocation or information rather than the expectations associated with the work itself. As Victoria, a coordinator for one of the Bustle’s local mutual aid groups, commented: ‘[they] had two, three retired social workers, [they] had trained counsellors that were volunteering, so [they] actually had the skills already there’ to serve people needing significant levels of support.

#### Stage 3

In Stage 3, restrictions were partially lifted, including for those most at risk in the ‘shielding’ category. This meant that there was a relative reduction in the level of need across all three communities. By this time, the local authorities had set up their operations and were delivering services, including working with charitable partners, across different community sectors. During Stage 3, the different types of relationships and interactions between the three different mutual aid groups and the public sector were most pronounced. Each mutual aid group seemed to play a different role in their local communities during this Stage, leading to groups going dormant, evolving into different entities, or honing and sometimes even scaling their ongoing service provision.

***The Green Isles – Stage 3.*** By Stage 3, the Green Isles group had gone into what could be considered a ‘hibernation’ mode, where they were present through their Facebook page, and open to requests, but there was very little group activity apparent during this period. The local authority continued to deliver services, although the major hub for the local authority began to wind up services. While the hub had initially operated from a sports hall, during this stage they moved some equipment to an office in the council buildings. Based on the level of requests the hub was receiving at this point, operating from those buildings was considered more efficient. The hub could potentially have been expanded again, if necessary since equipment had been gathered and processes had been developed over the previous two stages. Anna, part of the pre-existing ecosystem as a third sector focus group participant, discussed this shift when the Council ended up getting more involved in service delivery, ultimately improving the communication among all parties involved and streamlining service provision:

The local politics came into account and it wasn’t really an issue in the first couple of weeks, but then it was quite prominent … and that’s where the communications really stepped up because the Council was … [delayed] before they came in and the communications were really hard at the start, but thankfully … [the Council] actually manned the phone and because of these relationships it made the communication a lot easier.

Once the Council took over coordination, it allowed the volunteer mutual aid groups and the more formalized community groups to support community members where necessary, but in a more supplementary manner.

***The Dockyards – Stage 3***. The peri-urban Dockyards group maintained their activities during this period, although those activities were rebranded by the end of June 2020. Most of the services the group provided during Stage 2 were transitioned to the service portfolios of civil society organizations that had existed prior to the pandemic, especially as the mutual aid group became affected by volunteer fatigue and as some volunteers had to return to work as lockdown was relaxed. The end of June 2020 also marked the end of other services the group provided, while maintaining a few services under their organizational umbrella, primarily focused on addressing ongoing needs related to grieving and loss within the community. Yet, there were still antagonistic relationships between this mutual aid group and the local authority, as well as with some other statutory bodies. Mutual aid group members perceived these antagonistic dynamics to have negatively impacted service delivery within the community by disrupting ongoing initiatives that were providing aid.

***The Bustle – Stage 3***. The urban mutual aid group used this time to refine their services and to continue delivering services to those that they had been supporting throughout the lockdown. During this group’s meetings over the summer, some members warned that the mutual aid group should not frame this period as a ‘lifting of lockdown’ because, for many vulnerable individuals, their reality might not have changed to a great degree. As word spread about some of the community-based provision offered by neighbourhood-based mutual aid groups within the larger urban group, need apparently increased. For example, a food point set up by the group in Stage 1 had three people on its first day, but during Stage 3, it was visited by around 70 people per day, often coming from other neighbourhoods. In thinking about how much need there really was during this stage, and reflecting on cooperation during the crisis, Victoria revealed in her interview that:

The requirement for support is such that there’s plenty to go around … that partnership has made sure that it’s not about competing at all. For us there’s nothing in it, there’s not a competition because we’re not a constituted group where we access funding or we’re trying to say, you know, ‘we’ve supported more people than you’ve supported’ or anything like that. The motivation is just genuinely to support our own communities and to help. So, we don’t have that baggage or politics around it.

#### Stage 4

In this fourth stage, the needs of individuals within communities increased substantially from Stage 3 because restrictions were being re-introduced at this point. Each of the mutual aid groups started receiving more requests for help. Relationships between the formal and informal service providers began to revert to similar dynamics (in two of the three cases) as observed in Stage 1.

***The Green Isles – Stage 4***. During Stage 4, the rural island group came out of ‘hibernation’ and began posting more regularly with updates about information they had pulled together from local sources. Members of the Green Isles also shared that they started receiving more individual requests for support. Although, it is important to note that not all these requests were related to COVID-19 directly, but rather were from neighbours asking for help with tasks such as clearing driveways during a snowstorm. The group’s supplementary relationship with the state did not dramatically change during this stage because even though the local council-run support hub was scaled back during Stage 3, other more formal organizations stepped in to fill gaps and thus the mutual aid group helped provide a limited range of services, responding to niche requests that often were not directly related to COVID-19.

***The Dockyards – Stage 4***. By Stage 4, the peri-urban group had already concluded their response as a group explicitly formed to address community needs instigated and exacerbated by the pandemic. Members from the group were still meeting on a bi-weekly basis, but in a more informal manner than they did in Stages 1–3, and with the goal of linking ongoing community services and maintaining levels of collaboration inspired by the initial rapid response coordination. For example, in an interview with Flora, a member of the Dockyards, she said that the ‘ … response needs to be more about the medium to long-term and tapping into resources that are there and effective at the same time’. Several members expressed hope that collaboration would continue, but Callum predicted that ‘The 600 third sector organizations will move back into a competitive landscape as opposed to collaboration’ in an online activity exploring the future of public sector and civil society collaboration. Members of the peri-urban group had become aware that support would be required for an extended period, and perhaps longer than had initially been planned at the outset. In one of the discussion boards, Callum wrote:

… we need to think carefully about how we extract or exit our services, doing this in a dignified manner and one in which, nothing is missed, and no one is left in a precarious position. This will be very difficult. However, on the upside, this represents an opportunity for [community-led responses] to move into the gaps if our government/council won’t.

In our focus groups, many public sector, and formal third sector, officials described a desire to learn more about what the mutual aid groups might have done effectively, especially when they did not necessarily believe their work was constructive, so that lessons for more beneficial instead of adversarial partnership could be learned. Kyle represented this common view of focus group members saying:

One thing we feel like we’re continually learning more about and wanting to emphasize the importance of is working together between sectors, so statutory and community, volunteers as well is just more linking up … [we want to be] able to focus on where the greatest need might be, because if you’re working with established organizations who might know a bit more about that, then that can help.

Even though there was certainly some hesitancy around the work of mutual aid groups from the more formalized sector organizations and public sector bodies, particularly in this case and this stage, there was still a degree of good-will and a desire to develop a plan for improved working in the future that preserved dynamics that were positive. In many ways, it seemed that our focus group participants believed there were opportunities to engage mutual aid groups with already established, albeit more informal organizations, such as community enterprises.

***The Bustle – Stage 4***. During this time, when requests from specific postcode areas had reduced while apparent needs in other areas seemed to increase, the Bustle group streamlined their operations and, since they never ceased their service provision, they continued operating. Their operations were primarily collaborative, having established relationships with government actors and other formal civil society organizations; however, in group meetings, they still discussed opportunities to serve in more of an advocacy and activist role moving forward that could be seen as challenging the state and more formal voluntary organizations.

As the Bustle group evolved in Stage 4, so did the apparent concern about sustainability, service continuity, and local community partnerships. Some of the more local groups who had developed specialized services within the Bustle’s larger group began considering formally constituting as a mutual organization to protect their services and access more formal funding streams, which could further facilitate collaboration. In this case, they considered that it would also provide the group with more autonomy, since they would not have to be tied directly to other formal organizations for funding, but they would still be able to partner with these organizations.

## Discussion

While the schema of Young (2000) has been used as an analytical framework, the findings emerging from each case history also supported an understanding of what each of Young’s categories looked like ‘on the ground’ for the mutual aid groups. Firstly, the *complementary* form of relationship involved a level of collaborative working between the mutual aid group and their local authority, charities, and other formal organizations within civil society. For example, when the council or a charity in a local authority area received a request for help for a service that they did not undertake – like the delivery of a medical prescription – or simply did not have the capacity to respond, the organization would seek collaboration with the mutual aid group. Empirical evidence has shown that when civil society organizations complement already existing public provision (instead of acting as competitive *replacements* for these providers), increased levels of connectedness, well-being, and self-confidence among service users can be achieved (Calò et al. 2018). More recent research examining digital co-production of services during the pandemic has also demonstrated how this engagement improved public health and created public value (Zou and Zhao 2021).

The *supplementary* relationship, by contrast, was characterized by very few apparent collaborations between the mutual aid group and the state or other formal organizations. The mutual aid groups were supplementing public services in the (relative) absence of the state. For example, the mutual aid groups would provide food parcels to those in need, and support those who would not be eligible for access to state services.

The third type of relationship was one characterized by an *adversarial* relationship between the public sector and the mutual aid group. Within this context, local authorities and the mutual aid groups did not actively collaborate and at times even attempted to obstruct the other’s service delivery. For example, some mutual aid group members remarked that their local authority was slow because of their hierarchical structures and their bureaucracy, whereas some local authority workers expressed apprehension around the risks of personal protection for mutual aid group members as well as for those who requested support from the groups.

**Figure 2.**
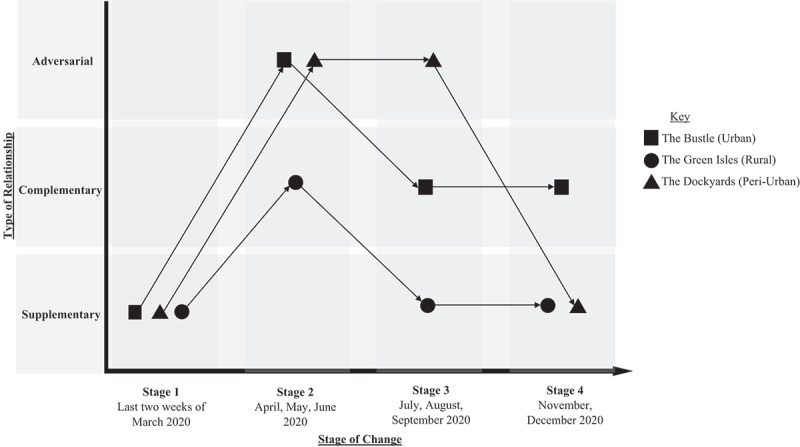
Mutual aid.

Young (2000) acknowledged that the three categories of relationship he put forward – complementary, supplementary, and adversarial – were not mutually exclusive, and were not fixed, but fluid: relationships between civil society organizations and the state can evolve and different roles will be performed over time. Movement between the different relationship positions is not a deliberate, agential choice: positions change over time according to national policies, immediate local circumstances, and the reactions that develop from these. These dynamics are key in the development of a nuanced understanding of how the mutual aid groups we studied evolved. A summary of the relationship changes is shown below in Figure 2, which illustrates the dynamic and changing nature of the relationships between mutual aid groups, the public sector, and other formal organizations over time.^3^

The Green Isles group was initially supplementary in their service delivery, providing services that were not being provided by the state or formal services. They soon moved to having a complementary relationship as service provision by the state and formal organizations were operationalized. This relationship became supplementary again as restrictions were lifted somewhat and members of the community could still request services that were not being delivered by the local authority or other organizations in the public service ecosystem. The group stayed supplementary through the final stage as they continued to operate in a niche area where other organizations did not. Group members perceived formal service provision as strong in their local area, indicating that most of the needs may have been addressed either by the state, by the other formal organizations on the island, or by neighbours and other connections in the community. However, the mutual aid group provided support upon request from individuals that were not being served by others: where there was need, there was seemingly little desire or requirement to collaborate with other services. When called upon, this group also shared their local knowledge of neighbourhoods and communities on the island so that public service organizations could more effectively respond to community needs and access citizens. This information sharing did not appear to foster meaningful ongoing collaboration around actual service delivery.

In contrast, from an initial supplementary position, the Dockyards group soon found themselves in an adversarial relationship with their local Council during Stage 2. The Council and mutual aid group seemed not to collaborate on service delivery, with each side seeing the other as, at times, obstructive. The Dockyards was, in a way, a collective of formal organizations that pooled existing resources and benefitted from a surge of new volunteers and additional philanthropic support for the newly developed group. These strong formal organizational connections may have developed because of slow and lacking state intervention in the local area. This lack of state intervention was documented at the time by the local and national press and media. As the Dockyards began to change at the beginning of Stage 3, their relationships with the state remained adversarial. As the group merged into another entity that was not itself delivering services, at the end of Stage 3 and into Stage 4, their relationship with other organizations and the state once again became supplementary to the plethora of other services that had then been established. This return to a supplementary relationship was also in part due to some volunteer fatigue associated with continuously ‘fighting’ to help the community and advocate for the needs of vulnerable individuals that they had identified, but failed to translate effectively for public sector prioritization and action (Grubb and Frederiksen 2021).

As with other groups the Bustle group initially had a supplementary relationship with the state and other formal services primarily due to their quick response. In Stage 2 this relationship developed into one that was more adversarial. Some initial frustrations with the local authority appeared within the group because of a perception that the state was still not providing what was needed by the community. It appeared that there was a sense among some mutual aid members that mutual aid was ‘better’ because they took more seriously the concerns of their communities and wanted to ‘get things done’. By Stage 3 and 4, the Bustle had established relationships within more organized civil society where partner organizations recognized their unique organizational characteristics and multiple organizations were more effectively able to collaborate in response to community needs.

Our findings thus lend nuance to the idea that mutual aid groups are primarily antagonistic at their root. We have evidenced here that, at times, these mutual aid groups were willing to step aside and allow collaboration between themselves and the public sector, for the betterment of their community during a crisis. This does not lessen the strength of mutual aid groups; on the contrary, it bolsters the remit of mutual aid which, as Springer (2020, 113) brings to our attention, is about cooperation which is as ‘equally, and, in point of fact, even more important in the perpetuation of life’ than competition.

Nevertheless, it is also true that these groups did develop antagonistic relationships with the state at times, particularly when it was considered the local authority were failing to provide adequate services to the community. In the local context of the relatively deprived Dockyards community, for example, it may have been the case that the antagonistic relationships that appeared during our study was the result of intensification of pre-existing antagonisms. Even for a group finding themselves in an antagonistic relationship, however, our findings show that these groups are not necessarily as adversarial at their core as they are often characterized (Dayson and Damm 2020); they can move through different relationships over time.

Moreover, the Dockyards continue to operate in their community as a conglomerate of formal organizations and so this development stemming from an adversarial relationship could be seen as the generative and positive change that North et al. (2020) suggested can happen. This finding particularly supports the call for multi-level and cross-sectoral solutions to the inherent systemic inequities that COVID-19 has exacerbated (Chakelian and Goodier 2020). While Shand et al. (2022, 14) state the importance of ‘collaborative governance arrangements and service delivery across multiple levels’ from a public service management perspective – it appears (at least within their limited number of case studies) that mutual aid and community-based initiatives are not considered an explicit part of their collaborative arrangements. From a public service logic standpoint (Osborne 2018) it is clear that civil society actors have an important role in public service provision. Shand et al. (2022) also evidence the tensions that public managers face in navigating between central, regional, and local levels. Our findings make explicit the contribution that mutual aid can make in time of crisis as part of a wider public sector response, but they also show a dissection of the shifting tensions over time. These findings may provide a clearer picture for public service managers as they navigate these tensions during future crises. More widely, these findings support the notion that there was a strengthening in community relations during the pandemic because of communities mobilizing themselves to respond to local needs (see Cook et al. 2020), to fill (albeit perhaps temporary) ‘institutional voids’ (Mair and Marti 2009).

## Conclusion

In closing, we recognize that there are several limitations of our study. This research was undertaken at an extremely critical and early point in the national lockdown, and we could not interview people face-to-face, nor visit the locations of the groups. Public services and civil society groups also had to completely reprioritize in the face of a public health crisis unprecedented (at least in the global north) in our lifetimes. While we would have preferred far more engagement with the software by the participants, and longer and deeper engagement from public officials other than during the focus groups, it should be recognized that our participants were in crisis mode and the level of engagement we were able to achieve, while sporadic, was not inconsiderable. Nevertheless, despite these challenges, we were able to arrange focus groups with 10 individuals from across eight different public and third sector organizations at the height of their response to the pandemic. These circumstances may have impacted upon the depth of our appreciation of context. However, we do know all of the locations and contexts relatively well and drew upon local knowledge when necessary.

A further limitation was the high drop-off rate for those signing up to the Recollective site then not completing activities. If these people had maintained their participation in the study, it may have led to the collection of more comprehensive data. Of those that did sign up to the study, there may well have been a bias towardss those that have a positive attitude towards the study, as well as those that were willing to express their voices. However, some of this potential positive bias was negated by our ethnographic approach, their publicly available conversations, and the negative comments expressed by participants.

While considering the role of public management in response to crisis, it is also important to interrogate how public service professionals, across all sectors, but particularly within the public sector, have responded. Through studying places where communities and civil society interfaced during a crisis, it is possible to explore the responses of public service professionals outside of government bureaucracy. Exploring different contexts, where the relationships between the public sector and civil society are entirely different, or even absent altogether, and research that further explores how mutual aid groups can pool local resources in times of need, would complement this study, providing a more holistic picture of local community responses to crisis and thus adding to our understanding in coping with future crises.

We set out to establish the different types of relationships between mutual aid groups and the public sector and explore how, and in what ways, such relationships adapted according to context, and over time. Our contribution here is in providing mutual aid groups, local public services, and local as well as national governments with the information that can help them to foresee, understand, and navigate through relationships at a time of crisis which, ultimately, can help to provide more effective service delivery for those most in need. We have shown that these mutual aid responses are relational (to the public sector response), and we have charted the dynamic relationship between the two. Our findings highlight the role that mutual aid can play in response to crises and offer potential areas of consideration for policymakers to better tap into a spirit of mutuality in times of crisis. Acknowledgement from national and local government of the role that mutual aid and the third sector more widely can play in their strategic and on-going responses to crises in the future will allow mutual aid groups and more formal service providers alike to help fill gaps in public service provision and best serve community needs.

## Supplementary Material

Supplemental Material

## Notes

1. At this point in the group sampling process, we did not sample according to the groups’ external dynamics (i.e. relationships with the state that were adversarial, complementary, or supplementary). We were interested in contextual and internal operational differences when selecting groups initially, and the differences in the types of relationships emerged through analysis in the latter stages of the research. This approach applied only to the sampling process and not the subsequent analytical work.

2. In using this framework to examine relationships between the state and civil society, we recognize that the ‘state’ is not simply an abstract macro-level structure but consists of a wide variety of different institutions with which the mutual aid groups interacted at different levels. Throughout the analysis we refer to the ‘state’ sometimes differentiating between various institutions and at other times using a collective reference depending on the framing of participants within the study.

3. Within each ‘stage’ the mutual aid groups were not typically exclusively displaying one type of relationship (i.e. in some respects actions were adversarial and in others they were supplementary within the broader public service ecosystem). This figure provides an overview that reflects the most prominent characterization of relationship between mutual aid groups and the state at each identified stage.
