# Supplementary material for: Relationships between community-led mutual aid groups and the state during the COVID-19 pandemic: complementary, supplementary, or adversarial?
Source: Public Manag Rev. 2022 Jun 6;26(2):313–33. doi: 10.1080/14719037.2022.2084769 (PMC11138323; doi:10.1080/14719037.2022.2084769)
Supplement: Supplemental Material [file RPXM_A_2084769_SM5811.zip › Appendix 2 Ethnographic Observations.docx]

| **Relationship with the State** | **Examples** |  |  |
| --- | --- | --- | --- |
| Supplementary | Photos uploaded by mutual aid group members dropping off prescriptions and groceries before local councils set up services for food and medicine delivery | Monitoring Facebook Group activity where the number of posts and interactions dramatically declined at later stages of the study, showing the increased role of the groups during the initial stages of the pandemic | Review of Slack messages that one mutual aid group used to communicate throughout much of their operations. This highlighted an awareness of existing services and indicated the establishment of channels to focus on activities that would serve needs not being addressed elsewhere |
|  |  |  |  |
| Complementary | Listening to local neighbourhood group meetings (often with 5-10 people joining virtually from formal and informal bodies) offering to collaborate and provide support based on their organizational or personal strengths | Attending webinars set up by formal third sector organizations which shared key insights from partnership working throughout the pandemic and touched on lessons learned from engaging mutual aid volunteers | Anecdotal stories from interviews (not quoted directly, but referenced in the findings section) about receiving calls from local council workers to help with individual requests from people that the council did not know how to access or serve |
| Adversarial | Observing mutual aid group meeting breakouts where members expressed interest in leaning into advocacy and using their position and spotlight as a group to prioritize social justice concerns around hunger and housing | Reflective notes from focus groups that commented on shifts in body language and facial expressions that indicated distaste and exasperation among public sector participants when more positive comments were made about mutual aid groups and their potential | Media coverage of various community responses combined with reactions and comments in Facebook Groups that expressed disagreement and frustration with more formal interventions, choices on spending money, and length of formal response times |

**Appendix 2: Examples of ethnographic observations, activities, and data points that informed overall analysis**
